# Risk factors for progression from *Clostridioides difficile* colonization (NAAT+/toxin–) to infection (toxin+) following symptomatic retesting

**DOI:** 10.1017/ice.2025.10377

**Published:** 2026-03

**Authors:** Sophia Chang, Nicholas Turner, Michael Yarrington, Deverick Anderson

**Affiliations:** 1 Duke University School of Medicine, Durham, NC, USA; 2 Division of Infectious Diseases, Duke University Medical Center, Durham, NC, USA; 3 Duke Center for Antimicrobial Stewardship and Infection Prevention, Durham, NC, USA

## Abstract

**Objective:**

To identify host and clinical risk factors contributing to the development of *Clostridioides difficile* infection (CDI) among colonized patients.

**Design:**

Retrospective, matched case-control study.

**Setting:**

Duke University Health System, including 3 hospitals and affiliated outpatient clinics.

**Participants:**

Adult patients who underwent ≥2 two-step *C. difficile* tests (nucleic acid amplification test (NAAT) followed by toxin enzyme immunoassay) between 03/15/2020–12/31/2023. Cases were patients with *C. difficile* colonization (NAAT+/toxin–) who converted to CDI (NAAT+/toxin+) within 90 days; controls were colonized patients who remained toxin-negative. Cases were matched to controls by date of index testing (±1 year).

**Methods:**

Data collection encompassed a 90-day “pre-exposure” period preceding index testing and a ≤ 90-day “exposure” period between index and repeat testing. Antibiotic use was stratified by risk for each period. Multivariate conditional logistic regression with forward selection was used to identify predictors of progression.

**Results:**

Among 2,212 colonized patients, 71 cases and 133 matched controls were identified. Several host and clinical characteristics were independently associated with progression to CDI in our multivariate model. Notably, high-risk antibiotic use across the pre-exposure and exposure periods was associated with greater odds of progression to CDI compared to other patterns of antibiotic use (adjusted odds ratio 2.70; *P* = .03).

**Conclusions:**

Sustained exposure to high-risk antibiotics was a strong predictor of the progression from *C. difficile* colonization to infection, underscoring the need for further research on longitudinal stewardship strategies for CDI prevention, particularly among patients previously identified as colonized.

## Introduction

*Clostridioides difficile* infection (CDI) is the leading cause of healthcare-associated diarrhea and the most common cause of healthcare-associated infection.^1,2^ Antibiotic exposure is an established risk factor for the development of CDI. Other risk factors for CDI include older age, chemotherapy, gastrointestinal surgery, frequent healthcare exposures, and comorbidities such as cardiac disease, chronic kidney disease, and solid organ transplantation (SOT).^3–5^

Importantly, the presence of *C. difficile* in the intestine does not necessitate disease. *C. difficile* colonization is defined by *C. difficile* detection in stool in the absence of clinical symptoms. Previous studies have demonstrated colonization rates ranging from 4% to 15% among healthy individuals, 3% to 21% among hospitalized patients, and 4% to 51% among long-term care facility residents.^6^ Colonization was initially thought to confer protection against symptomatic *C. difficile*; however, more recent research has indicated that it may increase the risk of CDI.^6–8^ A meta-analysis of 19 studies published in 2015 found that patients colonized with toxigenic *C. difficile* on admission had a relative risk of 5.86 (95% confidence interval [CI], 4.21–8.16) of developing CDI compared to non-colonized patients.^9^

A 2025 study by our group expanded on these results. Using data collected between 2020–2023, Turner et al. found that *C. difficile* colonization was associated with progression to CDI (adjusted hazard ratio [aHR], 5.06 [95% CI, 3.61–7.10]), particularly in the setting of high-risk antibiotic receipt (aHR, 15.71 [95% CI, 9.85–25.06]). Despite this association, the absolute rate of progression remained relatively low at 5% (95% CI, 4%–6%).^10^

Thus, among individuals colonized with *C. difficile*, only a subset progress to disease.^9,10^ The factors that determine why some convert while others remain asymptomatic are not well understood. Many studies have examined risk factors for CDI in general patient populations, but relatively few have focused specifically on patients with known colonization who develop active infection. The objective of this study was to identify host and clinical risk factors associated with the progression from *C. difficile* colonization to infection.

## Methods

### Study design and patient population

This retrospective, matched case-control study was conducted in the 3 hospitals and affiliated outpatient clinics within Duke University Health System (DUHS). Patients were evaluated for inclusion if they underwent ≥2 two-step stool tests for *C. difficile* between 15 March 2020 and 31 December 2023. The two-step algorithm consisted of a nucleic acid amplification test (NAAT; Cepheid Xpert) with reflex to toxin enzyme-linked immunosorbent assay (TechLab Quik Chek Complete) when NAAT positive. Testing was restricted to unformed stool specimens. Reports with a NAAT+/toxin–result included the comment: “Likely represents colonization with alternative cause of symptoms. Treatment generally not indicated unless clinical signs of severe or fulminant *C. difficile* disease. Consider Infectious Disease consult in questionable cases.”

*C. difficile* colonization was defined as the detection of *C. difficile* in the absence of toxin (NAAT+/toxin−), while infection was defined as the simultaneous detection of pathogen and toxin (NAAT+/toxin+). NAAT– subjects were excluded.^4,6,11^ The index test was defined as an initial NAAT+/toxin− result indicating *C. difficile* colonization. Cases were patients with colonization on index testing who progressed to infection within 90 days, as evidenced by a repeat test yielding a NAAT+/toxin+ result. Controls were colonized patients who did not progress to infection and remained toxin-negative on all subsequent testing within the same follow-up window. Patients were excluded if their index toxin-negative test was preceded by a toxin-positive result in the 90 days prior. For cases with >1 toxin-positive assay within the 90-day follow-up window, the test closest in time to the index was selected for inclusion. Cases were matched to controls at a minimum ratio of 1:1 and maximum ratio of 1:3 based on date of index testing (±1 year). Study subjects were identified via the Duke Enterprise Data Unified Content Explorer (DEDUCE) database.^12^ DUHS institutional review board reviewed the study design and determined it to be exempt with a waiver of consent for data collection.

### Definitions and data collection

**Figure 1.**
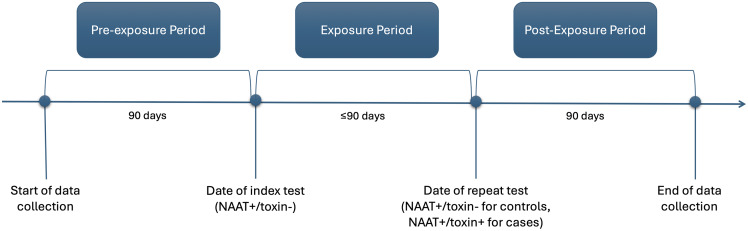
Temporal segmentation of patient data for assessing progression from *C. difficile* colonization to infection. NAAT, nucleic acid amplification test **(**Figure in accompanying digital file per submission guidelines).

To temporally distinguish risk factors influencing the progression from *C. difficile* colonization to infection, data collection for each patient was divided into three predefined intervals (Figure 1): a “pre-exposure” period, defined as the 90 days preceding the index colonization test; an “exposure” period, defined as the ≤90-day interval between the index test and the outcome event—the development of CDI for cases or the end of follow-up for controls; and a “post-exposure” period, defined as the 90 days following repeat testing.

All data were obtained through the DUHS electronic health record and DEDUCE database. For each patient, data were collected on demographics, outcomes, comorbidities (identified via *International Classification of Diseases, Tenth Revision* codes), recent healthcare exposures, and inpatient and outpatient antibiotic administrations. For systemic antibiotics, the class and duration of therapy were recorded. Admission details were collected for patients who were hospitalized at any time during the pre-exposure, exposure, and/or post-exposure periods. Outcomes studied included evidence of fulminant disease among cases, readmission in the post-exposure period, and death 1 year from the date of index testing.

### Antibiotic exposure classification

Given the central role antibiotics play in the pathogenesis of CDI, we applied a structured approach to represent patients’ time-varying antibiotic exposure profiles. Each systemic antibiotic administered to a patient was assigned a risk level. High-risk antibiotics were defined as clindamycin, fluoroquinolones, and third- or fourth-generation cephalosporins,^13^ while all other antibiotics were classified as low-risk. Each patient was then assigned a categorical exposure level for the pre-exposure period and the exposure period: no antibiotic exposure (“None”), only low-risk antibiotic exposure (“Low”), or any high-risk antibiotic exposure (“High”). From these two dimensions—risk level and timing—we classified patients’ cumulative antibiotic exposure profiles into nine mutually exclusive patterns: “None–None,” “None–Low,” “Low–None,” “Low–Low,” “High–None,” “None–High,” “High–Low,” “Low–High,” and “High–High.”

### Data analysis

Categorical variables were reported as frequencies and percentages and compared between cases and controls using Pearson’s chi-square test or Fisher’s exact test, as appropriate. Continuous variables were reported as medians with interquartile ranges (IQRs) and compared using the Wilcoxon rank-sum test. All pre-exposure and exposure period variables with a bivariate *P*-value ≤ .2 were evaluated for inclusion in multivariate modeling.

We utilized multivariate conditional logistic regression modeling to identify factors independently associated with the progression from *C. difficile* colonization to CDI. Covariates were selected for inclusion using a stepwise forward selection approach, with retention based on statistical significance (*P* ≤ .05) and adherence to event-per-parameter guidelines.^14^

Other antibiotic-related variables—such as number of antibiotic classes, duration of antibiotic therapy, and use of specific antibiotic classes—were excluded from the final models due to high collinearity with the variables characterizing patients’ cumulative antibiotic exposure profiles. Sensitivity analyses were conducted to ensure that their exclusion did not meaningfully alter the associations of interest.

Statistical analyses were performed using R, version 4.2.1

## Results

### Cohort characteristics

Among 2,212 patients with *C. difficile* colonization identified during the study period, 71 cases and 133 matched controls met criteria for inclusion. The cohort was 60% female with a median age of 62 [50–71] years. Most of the patients identified as White (63%), followed by Black (31%), with 6% identifying as another race. Demographics of case and control patients were generally similar (Table 1). The median time from index to repeat testing was 28 [17–51] days. Most patients underwent both index and repeat testing in an inpatient setting (66% and 65% respectively). Repeat testing in an inpatient setting was significantly more common among cases than controls (79% vs 57%; *P* = .003), while outpatient repeat testing was significantly less frequent in cases (18% vs 33%; *P* = .04).

**Table 1.** Host and clinical characteristics across pre-exposure and exposure periods

Variable	Cases (*n* = 71) *N* (%)	Controls (*n* = 133) *N* (%)	*P*-value
Demographics			
Age in years, median [IQR]	62 [49–70]	62 [50–71]	.87
Sex, female	39 (54.9)	83 (62.4)	.37
Race^a^			
Black or African-American	23 (32)	41 (31)	.94
White	41 (58)	87 (65)	.35
Other	7 (9.9)	5 (3.8)	.12
Ethnicity^b^			
Hispanic/Latino	3 (4.2)	2 (1.5)	.34
Non-Hispanic/Latino	66 (93)	128 (96)	.31
Host characteristics^c^			
Solid organ transplant	7 (9.9)	22 (17)	.28
Hematopoietic transplant	7 (9.9)	9 (6.8)	.61
Hospitalization within prior 12 months	43 (61)	106 (77)	.01
CCI Score, median [IQR]	7 [5–10]	7 [4–9]	.02
0–2	6 (8.5)	21 (16)	—
3–9	41 (58)	81 (61)	—
≥ 10	24 (34)	31 (23)	—
Comorbidities			
Heart failure	27 (38)	35 (26)	.12
Peripheral vascular disease	26 (37)	35 (26)	.19
Cerebrovascular disease	23 (32)	24 (18)	.03
Chronic pulmonary disease	21 (30)	43 (32)	.81
Diabetes mellitus with end-organ damage	22 (31)	48 (36)	.56
Chronic kidney disease	40 (56)	58 (44)	.11
Peptic ulcer disease	11 (15)	7 (5.3)	.03
Solid malignancy^d^	19 (27)	34 (26)	.99
Hematologic malignancy	11 (15)	13 (9.8)	.33
Pre-exposure period clinical characteristics			
Hospitalization	47 (66)	78 (59)	.36
Admission to the ICU	19 (27)	19 (14)	.05
Admission diagnosis^e^			
Infection	17 (24)	15 (11)	.03
Gastrointestinal disease	5 (7.0)	16 (12)	.38
Respiratory disease	11 (15)	9 (6.8)	.08
Cardiovascular disease	9 (13)	8 (6.0)	.17
Hematologic/oncologic disease	7 (9.9)	12 (9.0)	> .99
Days of hospitalization^f^, median [IQR]	9 [0–22]	1 [0–8]	.003
GI surgery	3 (4)	8 (6.0)	.75
GI procedure	7 (9.9)	16 (12)	.81
Chemotherapy	13 (18)	28 (21)	.78
Immunosuppression	38 (54)	71 (53)	> .99
Laxative use	41 (58)	67 (50)	.39
Exposure period clinical characteristics			
Hospitalization	57 (80)	97 (73)	.31
Admission to the ICU	17 (24)	15 (11)	.03
Admission diagnosis^e^			
Infection	12 (17)	28 (21)	.60
Gastrointestinal disease	14 (20)	30 (23)	.77
Respiratory disease	10 (14)	9 (6.8)	.14
Cardiovascular disease	16 (23)	6 (4.5)	.0002
Hematologic/oncologic disease	3 (4.2)	8 (6.0)	.75
Days of hospitalization^f^, median [IQR]	9 [3–20]	4 [0–10]	.001
GI surgery	2 (2.8)	0 (0)	.12
GI procedure	11 (15)	14 (11)	.42
Chemotherapy	9 (13)	16 (12)	> 0.99
Immunosuppression	38 (54)	59 (44)	.27
Laxative use	33 (46)	53 (40)	.44

IQR, interquartile range; CCI, Charlson Comorbidity Index; ICU, intensive care unit; GI, gastrointestinal.

^a^For cases, other race includes Asian (*n* = 2) and unknown (*n* = 5). For controls, other race includes American Indian/Alaska Native (*n* = 1), Asian (*n* = 3), and unknown (*n* = 1).

^b^Data were excluded for 2 cases and 3 controls where ethnicity was unknown.

^c^At time of index testing.

^d^Includes non-metastatic and metastatic disease.

^e^Admission diagnoses were not mutually exclusive; patients could be classified into more than 1 category. Additional admission diagnoses not shown in the table include renal disease, neurological disease, metabolic disorders, and miscellaneous.

^f^Includes subjects who were not hospitalized (0 days).

### Antibiotic characteristics

During the pre-exposure period, patients were prescribed a median of 2 [0–4] antibiotic classes, with nearly half (*n* = 100, 49%) receiving at least one high-risk antibiotic (Table 2). During the exposure period, patients were prescribed a median of 2 [0–3] antibiotic classes, with 89 (44%) patients receiving at least one high-risk antibiotic. In contrast, 55 (27%) patients in the pre-exposure period and 52 (25%) patients in the exposure period had no antibiotic exposure. Cases were more likely to receive high-risk antibiotics across both periods (High–High) compared to controls (*P* = .02).

**Table 2.** Antibiotic characteristics across pre-exposure and exposure periods

Variable	Cases (*n* = 71) *N* (%)	Controls (*n* = 133) *N* (%)	*P*-value
**Cumulative level of antibiotic exposure**			.06
None–None	13 (18)	19 (14)	—
None–Low	3 (4.2)	6 (4.5)	—
Low–None	1 (1.4)	10 (7.5)	—
Low–Low	3 (4.2)	20 (15)	—
High–None	1 (1.4)	8 (6.0)	—
None–High	5 (7.0)	9 (6.8)	—
High–Low	12 (17)	19 (14)	—
Low–High	5 (7.0)	10 (7.5)	—
High–High	28 (39)	32 (24)	—
**Pre-exposure period**			
Number of antibiotic classes^a^, median [IQR]	3 [0–5]	2 [0–4]	.08
0	21 (30)	34 (26)	—
1	2 (2.8)	25 (19)	—
2	10 (14)	21 (16)	—
3–4	16 (23)	32 (24)	—
≥5	22 (31)	21 (16)	—
High-risk antibiotic exposure^b^	41 (58)	59 (44)	.09
3rd/4th generation cephalosporins	30 (42)	47 (35)	—
Fluoroquinolones	21 (30)	32 (24)	—
Clindamycin	5 (7.0)	3 (2.3)	—
High-risk antibiotics DOT^c^, median [IQR]	2 [0-8]	0 [0-4]	.02
0	30 (42)	74 (56)	—
1–3	10 (14)	23 (17)	—
4–7	12 (17)	13 (9.8)	—
≥8	19 (27)	21 (16)	—
**Exposure period**			
Number of antibiotic classes^a^, median [IQR]	3 [1–4]	2 [0–3]	.005
0	15 (21)	37 (28)	—
1	7 (9.9)	25 (19)	—
2	13 (18)	34 (26)	—
3–4	23 (32)	28 (21)	—
≥5	13 (18)	9 (6.8)	—
High-risk antibiotic exposure^b^	38 (54)	51 (38)	.05
3rd/4th generation cephalosporins	30 (42)	38 (29)	—
Fluoroquinolones	16 (23)	21 (16)	—
Clindamycin	2 (2.8)	3 (2.3)	—
High-risk antibiotics DOT^c^, median [IQR]	1 [0–5.5]	0 [0-2]	.03
0	33 (46)	82 (62)	—
1–3	16 (23)	23 (17)	—
4–7	10 (14)	12 (9.0)	—
≥8	12 (17)	16 (12)	—
Presumptive treatment^d^	28 (39)	40 (30)	.23
Oral vancomycin	28 (39)	38 (29)	—
Fidaxomicin	3 (4.2)	5 (3.8)	—

IQR, interquartile range; DOT, duration of therapy.

^a^Antibiotics were divided into the following classes: 3rd/4th generation cephalosporins; fluoroquinolones; clindamycin; aminoglycosides; 1st/2nd/5th generation cephalosporins; macrolides; penicillins; beta-lactamase inhibitor combinations; sulfonamides; carbapenems; IV vancomycin; tetracyclines; metronidazole; and miscellaneous (fosfomycin, nitrofurantoin, methenamine, linezolid, tedizolid, dapsone, daptomycin, isoniazid, rifampin, and rifaximin).

^b^The summed antibiotic count exceeds the number of patients exposed, as some individuals received multiple classes.

^c^Measured in days. Different antibiotic classes administered on the same day were each counted as separate antibiotic days.

^d^Administration of oral vancomycin or fidaxomicin at any time during the exposure period was considered presumptive treatment. The summed antibiotic count exceeds the number of patients treated presumptively, as some individuals received both antibiotics.

**Table 3.** Multivariate conditional logistic regression model for the progression from *C. difficile* colonization to infection

Variable	Adjusted odds ratio (95% Confidence Interval); *P*-value
High–High antibiotic exposure pattern	2.70 (1.07–6.77); .03
Charlson Comorbidity Index score^a^	
0–2	Reference
3–9	2.21 (0.67–7.33); .19
≥10	6.13 (1.50–24.95); .01
Peptic ulcer disease	4.99 (1.25–19.99); .02
Solid organ transplant	0.24 (0.06–0.96); .04
Hospitalization within preceding 12 months	0.15 (0.06–0.39); .0001
Cardiovascular admission^b^ during exposure period	6.46 (1.88–22.14); .003

^a^At time of index testing.

^b^Identified by searching the admission diagnosis text for any of the following keywords: myocardial, heart, cardiac, cardiomyopathy, cardiovascular, vascular, circulatory, ECMO, or arrhythmia.

### Outcomes

In the post-exposure period, readmissions occurred in nearly half of patients (*n* = 69, 34%) with no significant differences between cases and controls. A total of 62 (87%) cases received treatment, and oral vancomycin was the most frequently administered therapy (*n* = 58, 82%). A small proportion of cases did not receive therapy, for reasons that were not captured. Evidence of fulminant disease among cases was rare; only 2 cases (3%) required rectal vancomycin, and 1 case (1%) underwent a colectomy. Mortality at 1 year was similar between cases and controls at 26.76% and 27.07% respectively.

### Risk factors for the progression from *C. difficile* colonization to infection

In bivariate analyses, several variables differed significantly between cases and controls (Table 1). Cases were more likely to have cerebrovascular disease, peptic ulcer disease (PUD), and a higher baseline Charlson Comorbidity Index (CCI) score.^15^ They were also less likely to have a history of hospitalization in the 12 months preceding colonization. In terms of clinical risk factors, cases experienced higher rates of intensive care unit (ICU) admission, longer hospital stays, and more cardiovascular-related admissions in the exposure period. Cases also received a greater number of antibiotic classes during the exposure period but had longer durations of high-risk antibiotic therapy in both time periods (Table 2). High-risk antibiotic use during the exposure period and across both periods was significantly more common among cases.

In our final multivariate model, high-risk antibiotic use across both the pre-exposure and exposure period was associated with progression to CDI in comparison to all other exposure patterns (adjusted odds ratio (aOR), 2.70 [95% CI, 1.07–6.77]; *P* = .03). Several non-antibiotic factors were also found to be contributing to or protective against CDI, including a CCI score ≥ 10, PUD, SOT, a cardiovascular-related admission during the exposure period, and a history of hospitalization within 12 months of index testing.

As a sensitivity analysis, an interaction model incorporating pre-exposure and exposure-period antibiotic risk profiles was constructed to assess whether sequential antibiotic use conferred greater risk than isolated use; to account for the temporal sequence of susceptibility; and to distinguish between isolated, sustained, and mixed-risk patterns (Supplementary Data Table 1). Patients who received low-risk followed by high-risk antibiotics (Low–High) demonstrated higher odds of CDI progression (aOR, 134.57; *P* = .02). Those with sustained high-risk antibiotic use (High–High) exhibited the greatest risk (aOR, 316.63; *P* = .01). A likelihood ratio test comparing the full interaction model to a nested main-effects-only model with identical covariates revealed that the addition of interaction terms improved model fit (χ^2^ = 11.12 on 4 df, *P* = .03), though findings were interpreted with caution given model instability and wide confidence intervals.

In an additional sensitivity analysis excluding controls who received presumptive treatment, results were consistent with the primary model (Supplementary Data Table 2). Sustained high-risk antibiotic use across the pre-exposure and exposure periods remained strongly associated with progression from colonization to infection (aOR 6.08, [95% CI, 1.80–20.6]; *P* = .004). Associations between non-antibiotic covariates were similar in direction and magnitude to those observed in the main analysis, though estimates were imprecise given reduced sample size.

To explore the unexpected protective association of prior hospitalization, we conducted a series of post hoc analyses. One hypothesis was that patients with more prior hospitalizations received more proactive intervention. However, when we analyzed rates of presumptive treatment, we found no meaningful differences across hospitalization strata. Differential treatment patterns alone are therefore unlikely to account for the observed association. We also explored whether patients with prior hospitalizations were less likely to receive high-risk antibiotics as a result of increased monitoring and antibiotic stewardship. Contrary to this hypothesis, patients with a history of recent hospitalization were significantly more likely to receive high-risk antibiotics during both the pre-exposure (χ² = 10.9, df = 1, *P* = .001) and exposure periods (χ² = 11.15, df = 1, *P* = .001). Thus, further investigation and validation of this finding is required.

## Discussion

Our study provides novel information about potential risk factors for the development of CDI among patients previously identified with *C. difficile* colonization.^10,16^ Results of our multivariate model and sensitivity analyses revealed a potential dose-dependent trend in which earlier, prolonged, and higher-risk antibiotic exposure conferred progressively greater odds of CDI. These observations suggest that both the timing and class of antibiotic therapy contribute to CDI pathogenesis among colonized patients, with sequential exposure to higher-risk agents likely compounding microbiome disruption and contributing to loss of colonization resistance. These findings also underscore the importance of antibiotic stewardship not only after colonization is identified but also during preceding healthcare encounters, when early antimicrobial decisions may influence downstream infection risk.

In addition, several non-antibiotic factors emerged as independent predictors of progression to CDI. Consistent with prior research, hospitalization during the exposure period for cardiovascular-related conditions was strongly associated with conversion^17,18^; for example, Mamic et al. observed that patients admitted for heart failure had a 13% elevated risk of developing CDI.^17^ This risk may be related to increased systemic inflammation and sympathetic activity, and/or volume depletion and demand mismatch.^18^ This association may also reflect the increased clinical severity and complexity of cardiovascular admissions. Among all hospitalized patients in our cohort, cardiovascular admissions were 3.25-times (95% CI, 1.09–9.40) more likely to involve intensive care compared to non-cardiovascular admissions.

Additional independent predictors of progression in our study included a CCI score ≥ 10 and the presence of PUD at the time of index testing. A higher CCI score likely reflects greater systemic vulnerability due to disease burden and treatment, which may lower the threshold for colonization to transform into clinically significant infection.^19,20^ PUD may contribute further risk by compromising mucosal integrity, though its specific role in CDI pathogenesis warrants further investigation.^21^ Interestingly, in contrast to most existing literature, SOT was independently associated with a lower risk of progression in our study. The number of SOT recipients in our cohort was relatively small (*n* = 29, 14%), and the observed association may reflect unmeasured confounding, limited power, or center-specific practices. A 2020 retrospective cohort study reported relatively low rates of progression from colonization to infection in SOT cohorts, possibly reflecting early intervention and aggressive infection prevention protocols.^22^ While these findings offer a plausible explanation for our observed association, they are exceptions rather than the norm in the literature. Further investigation is needed to clarify whether certain clinical protocols in transplant care may influence risk of progression.

Several risk factors previously linked to CDI were not independent predictors in our cohort. Age, laxatives, chemotherapeutics, and immunosuppressants did not differ significantly between cases and controls.^3–5^ Presumptive treatment was also not associated with a lower risk of subsequent toxin positivity, despite being previously reported as protective in a similar study^16^ and by prior analyses conducted by our group.^10^

Healthcare exposure patterns in our study revealed a complex relationship with CDI risk. Most notably, a history of hospital admission in the preceding 12 months was associated with a strong protective effect in both bivariate and multivariate analyses—contrary to established literature that identifies prior hospitalization as a risk factor.^3–5^ Additionally, residence in skilled nursing facilities and other healthcare settings prior to admission were not significantly different among cases and controls.^4,5^ It is conceivable that these and the preceding patient-level risk factors described above may actually be more associated with colonization (which all included patients had by definition) as opposed to progression from colonization, but further investigation of this hypothesis will be required. Prolonged hospitalization and ICU admission were statistically associated with progression in bivariate analyses, suggesting that certain forms of healthcare utilization remain important risk factors for CDI.

Our study has limitations. While we operationalized NAAT+/toxin– as a proxy for colonization, some of these controls may have had early or partially treated CDI. As testing was limited to patients with unformed stool, the study cohort likely included a mixture of true infection and colonization with alternate causes of diarrhea. This distinction is a known limitation of NAAT-based testing algorithms and of retrospective designs relying on clinical, rather than surveillance, sampling. Further reflecting the diagnostic uncertainty inherent to this group, approximately one-third of controls received CDI-directed therapy. However, the robustness of results in our sensitivity analysis excluding presumptively treated controls suggests that this potential misclassification did not materially affect our conclusions. Additional limitations include data being restricted to encounters, prescriptions, and laboratory testing documented within DUHS. Moreover, while we adjusted for several known risk factors, the possibility of residual confounding from unmeasured or imperfectly measured variables remains. Repeat testing intervals were not standardized, and despite excluding periods >90 days, timing variability may have influenced exposure assessment. We were unable to fully adjust for the severity of acute illness that may influence CDI pathogenesis. Microbiological data such as *C. difficile* strain type, which could affect virulence, were also not available. Differences in clinician testing practices, care environments, or social determinants of health may have also influenced results in ways not fully captured in our data set. Next, the sample size of our cohort likely limited statistical power and our ability to detect associations for less common exposures or outcomes. Finally, the generalizability of our findings may be limited by the nature of the study setting, study population, and retrospective study design.

This retrospective, matched case-control study highlights the differential roles host factors and clinical characteristics play in the progression to CDI among patients identified with *C. difficile* colonization. We found that high-risk antibiotic use before and after the identification of colonization significantly influenced disease progression, reinforcing the need for vigilant antimicrobial stewardship across the continuum of care—not only after colonization is detected but also during routine healthcare encounters in at-risk populations. The period following colonization represents a particularly critical window during which avoiding high-risk antibiotics may offer the greatest opportunity for targeted stewardship interventions. Further prospective research is needed to refine risk stratification and identify colonized individuals most likely to benefit from early intervention.

## Supporting information

Chang et al. supplementary material Chang et al. supplementary material

## Supplementary material

The supplementary material for this article can be found at https://doi.org/10.1017/ice.2025.10377
