# Supplementary material for: Risk factors for progression from Clostridioides difficile colonization (NAAT+/toxin–) to infection (toxin+) following symptomatic retesting
Source: Infect Control Hosp Epidemiol. 2025 Dec 19;47(3):265–71. doi: 10.1017/ice.2025.10377 (PMC12932920; doi:10.1017/ice.2025.10377)
Supplement: Chang et al. supplementary material [file S0899823X25103772sup001.docx]

**Supplementary Data**

**Risk factors for progression from *Clostridioides difficile* colonization (NAAT+/toxin–) to infection (toxin+) following symptomatic retesting**

**Table S1. Multivariable Conditional Logistic Regression Model Incorporating Interaction Term.**

| **Variable** | **Adjusted Odds Ratio (95% Confidence Interval); *P*-value** |
| --- | --- |
| Antibiotic exposure: pre-exposure x exposure interaction | —; 0.03 |
| None–None | Reference |
| Low–Low | 8.23 (0.22–309.l7); 0.25 |
| High–Low | 46.68 (0.80–2,708); 0.06 |
| Low–High | 134.57 (2.18–8,324); 0.02 |
| High–High | 316.63 (4.67–21,470); 0.01 |
| Charlson Comorbidity Index score¹ |  |
| 0–2 | Reference |
| 3–9 | 2.21 (0.67–7.33); 0.19 |
| ≥10 | 6.13 (1.50–24.95); 0.01 |
| Peptic ulcer disease | 4.99 (1.25–19.99); 0.02 |
| Solid organ transplant | 0.24 (0.06–0.96); 0.04 |
| Hospitalization within preceding 12 months | 0.15 (0.06–0.39); 0.0001 |
| Cardiovascular admission² during exposure period | 6.46 (1.88–22.14); 0.003 |

¹At time of index testing.

² Identified by searching the admission diagnosis text for any of the following keywords: myocardial, heart, cardiac, cardiomyopathy, cardiovascular, vascular, circulatory, ECMO, or arrhythmia.

**Table S2. Sensitivity Analysis Excluding NAAT+/toxin– Patients Who Received Presumptive Treatment.**

| **Variable** | **Adjusted Odds Ratio (95% Confidence Interval); *P*-value** |
| --- | --- |
| High-High antibiotic exposure pattern | 6.08 (1.80–20.6); **0.004** |
| Charlson Comorbidity Index score¹ |  |
| 0–2 | Reference |
| 3–9 | 1.87 (0.39–9.00); 0.43 |
| ≥10 | 5.37 (0.87–32.95); 0.07 |
| Peptic ulcer disease | 12.22 (1.58–94.30); **0.02** |
| Solid organ transplant | 0.19 (0.04–0.96); **0.05** |
| Hospitalization within preceding 12 months | 0.11 (0.03–0.38); **0.001** |
| Cardiovascular admission² during exposure period | 8.23 (1.76–38.43); **0.01** |

¹At time of index testing.

² Identified by searching the admission diagnosis text for any of the following keywords: myocardial, heart, cardiac, cardiomyopathy, cardiovascular, vascular, circulatory, ECMO, or arrhythmia.
